# Polarization-controlled metasurface for simultaneous holographic display and three-dimensional depth perception

**DOI:** 10.1515/nanoph-2024-0509

**Published:** 2025-01-17

**Authors:** Shuhan Guo, Yifan Shao, Junjie Zhan, Jiaqi Yu, Yubo Wang, Pankaj K. Choudhury, Hugo E. Hernandez-Figueroa, Yungui Ma

**Affiliations:** State Key Lab of Modern Optical Instrumentation, Centre for Optical and Electromagnetic Research, College of Optical Science and Engineering, International Research Center for Advanced Photonics (Haining Campus), 528788 Zhejiang University, Hangzhou 310058, China; Department of Communications, School of Electrical and Computer Engineering (FEEC), University of Campinas (UNICAMP), Campinas 13083-852, São Paulo, Brazil

**Keywords:** metasurfaces, structured light projection, three-dimensional reconstruction, holography, Dammann gratings, polarization multiplexing

## Abstract

Simultaneous optical display and depth perception are crucial in many intelligent technologies but are usually realized by separate bulky systems unfriendly to integration. Metasurfaces, artificial two-dimensional optical surfaces with strong light–matter interaction capabilities at deep subwavelength scales, offer a promising approach for manufacturing highly integrated optical devices performing various complex functions. In this work, we report a polarization-multiplexed metasurface that can functionally switch between holographic display and Dammann gratings. By tailoring the incidence polarization, the metasurface can display high-quality holographic images in the Fresnel region or project a uniform spot cloud nearly covering the entire 180° × 180° transmissive space. For the latter, a projection and three-dimensional (3D) reconstruction experiment is conducted to elaborate the potential in retrieving 3D complex spatial information. The current results provide a prominent way to manufacture lightweight and highly-integrated comprehensive imaging systems especially vital for cutting-edge intelligent visual technologies.

## 1 Introduction

Optical display and depth perception are key functions in many intelligent technologies wherever image guiding in a real-time 3D scenario operation is necessary. Particularly, in augmented/mixed reality (AR/MR) devices [1], [2], in order to generate an immersive experience, it is generally required to project certain virtual scenes on the real world, with the help of the depth perception system perceiving the environment and ensuring the correct projection of virtual images. For instance, in intelligent neuronavigation operations, the head-mounted MR devices can provide doctors with correct projection of the tumour models under the collective work of both optical display and depth perception systems, thus reducing the inconvenience of traditional monitors [3]. Similar display-sensing technologies have been excised in intelligent vehicles, where AR head-up display and light detection and ranging system have become the standard assemblies to provide comprehensive navigation information [4], [5]. However, these basic functions are currently achieved using separate, complex, and bulky systems in commercial products, impeding progress toward integration and miniaturization.

Two-dimensional nanostructured metasurfaces [6] are capable of manipulating light–matter interaction at deep subwavelength scales by precise and flexible control over the phase [7], [8], amplitude [9], [10], polarization [8], [11], [12], etc. These have led to varieties of promising applications in holography [13], [14], beam steering [15], [16], imaging and sensing [17], [18], etc. More specifically, compared to traditional diffractive optical elements (DOEs) such as spatial light modulator (SLM) [19], meta-holography enables more complex and precise light field manipulation that are instrumental for realizing compact 3D [20], [21], [22] or full-color display [23], [24], optical encryption [25], [26], and data storage [27], [28]. On the other side, compared to other passive depth sensing techniques [5], [29], [30], [31], [32], [33], uniform structured point cloud projected by Dammann gratings [34] enables active illumination and depth ranging over multiple points with a single shot. Employing metasurfaces, one could greatly improve the key performance parameters of Dammann gratings like field of view (FOV), attributed to the subwavelength pixel engineering [35], [36], [37], which has established a plaform to manufacture on-chip optoelectronic devices for versatile 3D sensing purposes [38], [39], [40], [41]. By encoding the phase profiles of hologram and Dammann gratings into one metasurface, the integration of optical display and depth perception has been realized recently [42]. However, due to the position multiplexing method, it suffers large efficiency penalty for both functions, and the periodic arrangement of the displayed images also limits the application scenarios.

**Figure 1:**
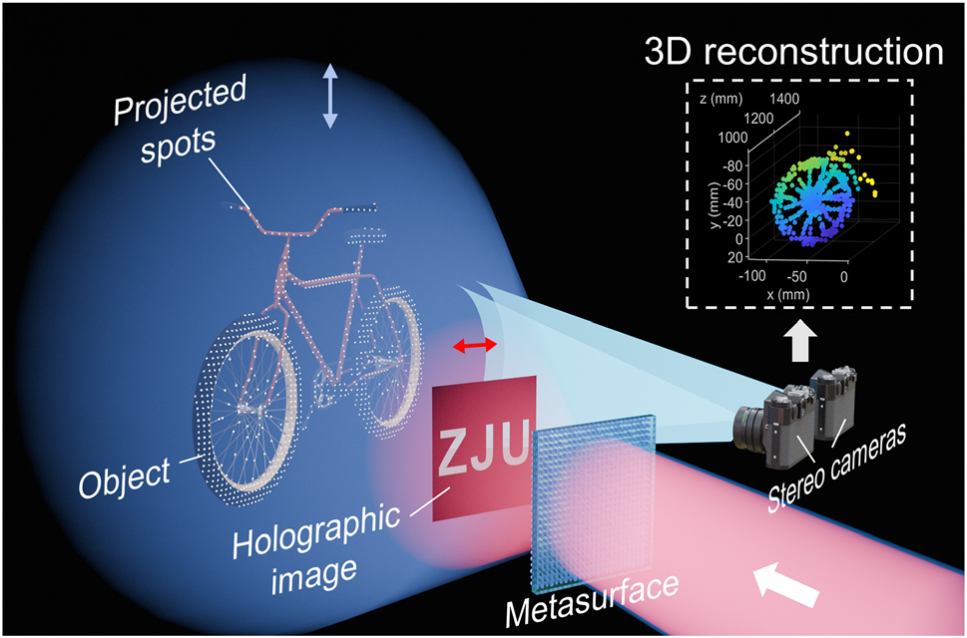
Schematic of the polarization-multiplexed metasurface. For the *x*-polarized light (the red beam), holographic images are formed in the Fresnel region. For the *y*-polarized light (the blue beam), uniform diffraction orders are generated to provide spot cloud illumination, nearly covering the entire transmissive space. By setting up a stereo system, 3D reconstruction of the measured objects can be completed. Insets: example of a reconstructed wheel model.

In this work, we propose a polarization-multiplexed metasurface that can integrate meta-holography with Dammann gratings in a high compact manner. Compared to other multiplexing methods, such as spatial/wavelength multiplexing, the crosstalk of the two functions can be minimized due to the independent control of phase profile for two orthogonal polarization states [28], [43], while the holographic image can be displayed independently without being affected by the periodic phase profile required by Dammann gratings. As shown in Figure 1, a high-quality holographic image is generated in the Fresnel region, meanwhile, a uniform spot array is projected into the far field, with a FOV of nearly 180° × 180°. As a proof-of-concept for application, by setting up a stereo system, we further conduct a projection and 3D reconstruction experiment of different objects to elaborate its potential in 3D spatial sensing. Consequently, such a multifunctional metasurface design is a promising approach for future optical devices with high compactness and scalability. It paves the way for applications of optical display and spatial sensing, which are especially required in cutting-edge technologies such as AR/MR techniques and intelligent driving.

## 2 Design method and simulation results

**Figure 2:**
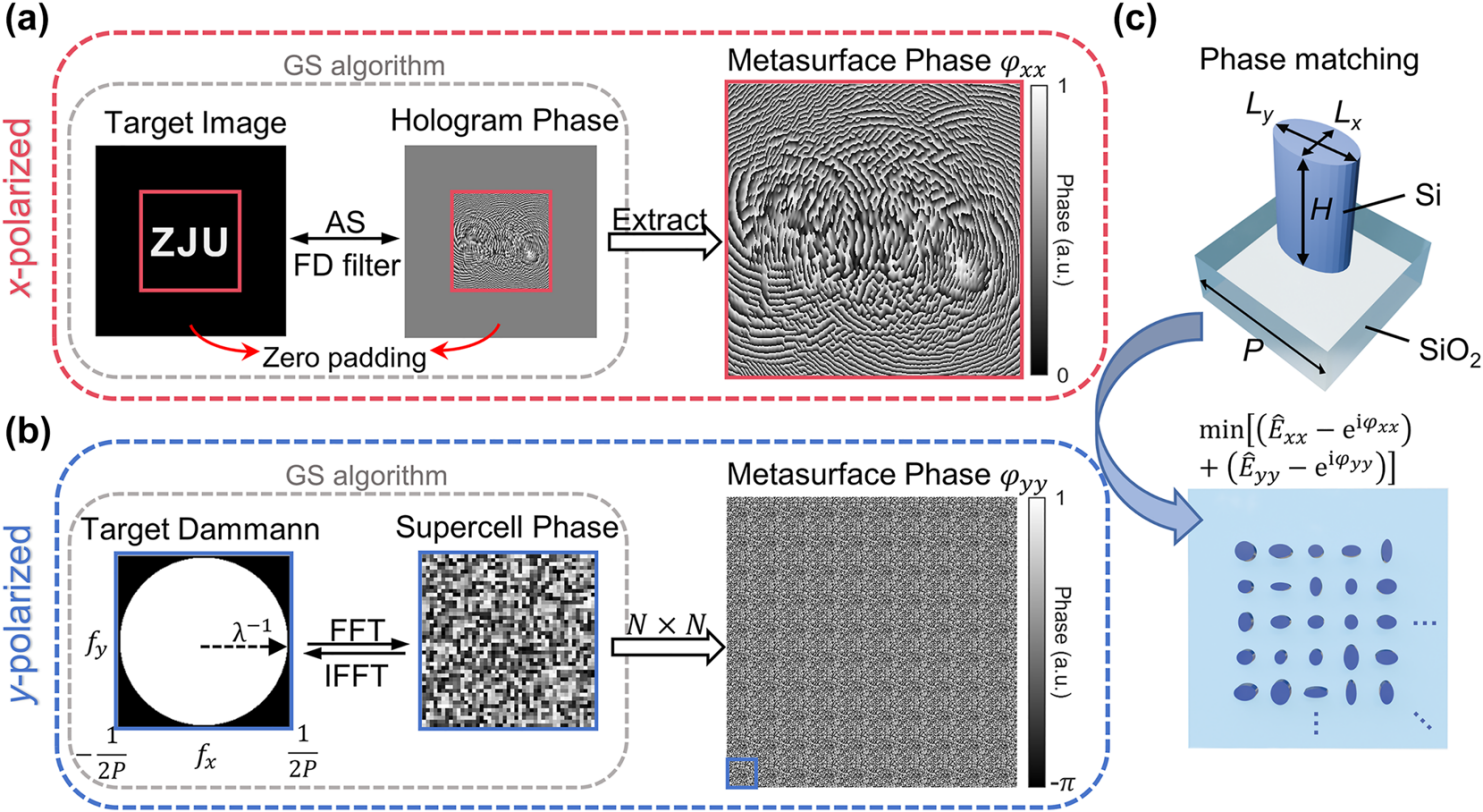
Schematic Flowchart of the optimization algorithm. (a) Optimization of the target holographic images in the *x*-polarization state using the GS and AS algorithms. The phase profile of the whole metasurface is retrieved as a hologram (outlined in red border). (b) Optimization of the target Dammann gratings in the *y*-polarization state using the GS algorithm and FFT. Only the phase profile of a single supercell is needed to retrieve here due to the periodicity (outlined in blue border). (c) Schematic of the structure of a meta-atom and phase matching condition. The unit-cell structure is composed of a silicon nanopillar with varying length *Lx/Ly* on a silica substrate. For each pixel, the chosen structure should satisfy the minimum deviation of complex amplitude from the target one for both polarization states.

The multifunctional metasurface incorporates two basic functions: meta-holography and Dammann gratings, achieved by imparting distinct phases on orthogonal linear polarization states. Figure 2 schematically shows the optimization algorithm. For the *x*-polarized light, a hologram is encoded using the Gerchberg–Saxton (GS) algorithm [44] and angular spectrum (AS) algorithm [45], as Figure 2(a) depicts. The overall hologram contains 450 × 450 pixels, and the distance *z* from the hologram to the image plane is set to be 380 μm. Zero padding and a low-pass spatial frequency filter are employed to suppress frequency aliasing and to optimize for the best reconstructed image quality under the condition $\left(f_{x}^{2}+f_{y}^{2}\right)\leq {\left(\frac{NA}{\lambda }\right)}^{2}$ . Here *f*_*x*_ and *f*_*y*_ are, respectively, the spatial frequency components along the *x*- and *y*-axes, *λ* is the operating wavelength, and NA (= 0.65) is the numerical aperture of the objective lens (40×) being used in experiments. The Pearson correlation coefficient is used as the figure-of-merit (FoM) here to retrieve the phase profile of hologram, which is defined as $\rho \left(T,R\right)=\frac{cov\left(T,R\right)}{\sigma_{T}\cdot \sigma_{R}}$ , where *T* and *R* represent the amplitude distributions of the target and reconstructed images, respectively, $cov\left(T,R\right)$ is the covariance of *T* and *R*, and σ_*T*_ and σ_*R*_ are the respective standard deviation of the same.

For the *y*-polarized light, a spot cloud is produced in the far field, with uniform intensity and nearly 180° FOV. The corresponding phase profile of the proposed metasurface comprises 10 × 10 supercells, and each supercell contains 45 × 45 pixels. The analysis is conducted on two aspects: the far-field diffraction patterns generated by one single supercell and the collective effect of periodic arrangement via interference.

For the supercell design, the GS algorithm is used to retrieve the phase profile, as shown in Figure 2(b). The sampling frequency is limited by $\left|f_{x,y}\right|\leq \frac{1}{2P}$ , where *P* refers to the pixel pitch, while the maximum spatial frequency of transmissive waves in free space is $\frac{1}{\lambda }$ . To fully recognize all the frequency components, *P* is set to 520 nm to satisfy the condition of $\frac{1}{\lambda }\leq \frac{1}{2P}$ at an operating wavelength of 1,064 nm, according to Nyquist sampling theorem. The target amplitude distribution is shown inside the blue color border, where the region inside the circle with a radius of $\frac{1}{\lambda }$ is set as 1 and elsewhere to 0. In this way, all propagating waves in the transmissive space are optimized for uniform intensity.

After applying the periodic arrangement, the phase profile of metasurface can be obtained. The corresponding propagation angle of the (*p*th, *q*th) diffraction order is $\left(\arcsin\left(\frac{p\lambda }{d}\right),\arcsin\left(\frac{q\lambda }{d}\right)\right)$ , derived from the grating equation, where *d* refers to the period of the supercell. Here the FoM is defined as $FoM=\left(1-DE\right)+RMSE$ to consider both the diffraction efficiency (DE) and the root-mean-square error (RMSE). The DE represents the proportion of total intensity of target diffraction orders, defined as $DE=\overset{M}{\underset{i=1}{\sum }}I_{i}$ , and the RMSE represents the uniformity of target diffraction orders, expressed as $RMSE=\frac{1}{1/M}\overset{M}{\underset{i=1}{\sum }}\sqrt{\frac{{\left(I_{i}-1/M\right)}^{2}}{M}}$ , with *M* being the total number of target orders and *I*_*i*_ being the intensity of the *i*th order normalized to that of the incident light. Figure S1 (Supplementary Material) shows the FoM as a function of iteration.

To implement the phase profiles on both polarization states, we consider the unit cell comprising an elliptic silicon nanorod on a fused silica substrate, as shown in Figure 2(c). For each unit-cell, the period *P* is fixed at 520 nm, and the height *H* of nanorod is fixed at 600 nm. Using the CST Studio Suite, nanostructures of different phases can be obtained by scanning the length along the *x*- and *y*-axes, as shown in Figure S2 (Supplementary Material). After that, structures with different *L*_*x*_ and *L*_*y*_ ranging from 140 to 400 nm are retrieved for the best match of both the *x*- and *y*-polarized phase profiles. For every pixel, the matching method is described as $\min\left[\left({\hat{E}}_{xx}-{\text{e}}^{\text{i}\varphi_{xx}}\right)+\left({\hat{E}}_{yy}-{\text{e}}^{\text{i}\varphi_{yy}}\right)\right]$ , where ${\hat{E}}_{xx,yy}$ refers to the *x*- and *y*-polarized complex amplitude of a certain unit-cell, and *φ*_*xx*,*yy*_ refers to the target *x*- and *y*-polarized phase. Figure S3 (Supplementary Material) exhibits the complex amplitude deviations of metasurface brought by phase matching. As a result, a holographic image with a correlation coefficient of ∼0.94 is generated in the image plane. Meanwhile, over 1.5 K spots are projected into the far field, nearly covering the full transmissive space, with a fairly good numerical DE of 98.3 % and an RMSE of 7.1 %. Table S1 (Supplementary Material) further shows the comparison of numerical performance of both functions among when encoding only the hologram, only the Dammann gratings, both functions using only position multiplexing (reference [42]), and both functions using polarization and position multiplexing (this work). The results show a high performance of both functions using our proposed method with a rather low total efficiency penalty of 0.028, much better than that when using only position multiplexing (0.276).

## 3 Experimental results

### 3.1 Experimental results of meta-holography and spot cloud projection

**Figure 3:**
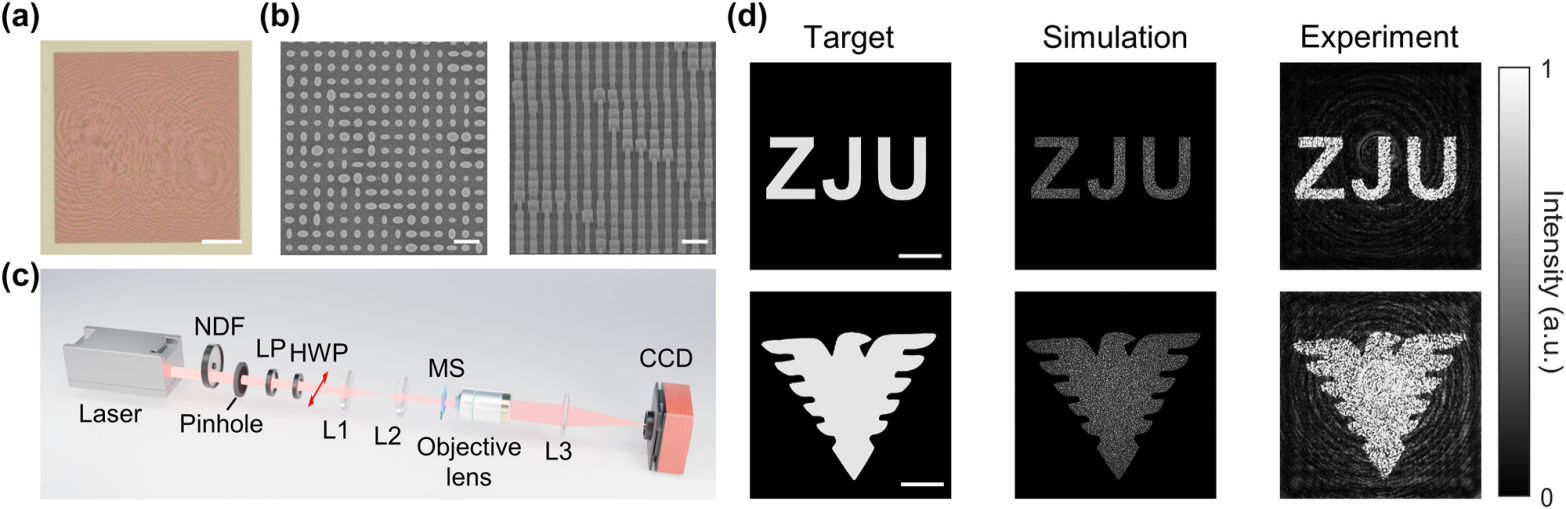
Experimental and numerical results of the holographic images. (a) Optical micrograph of sample 1; scale bar, 50 μm. (b) SEM images of sample 1; scale bar, 1 μm. (c) The optical setup for capturing the holographic images. Laser, 1064 nm, 200 mW; NDF, neutral density filter; LP, linear polarizer; Pinhole (500 μm); HWP, half-wave plate, is rotated to produce the *x*-polarized light (red arrow); L1, lens (*f* = 100 mm); L2, lens (*f* = 50 mm); MS, metasurface; Objective lens, 40×, NA = 0.65; L3, tube lens (*f* = 180 mm); CCD, short-wave infrared CCD (TekWin SC640). (d) The normalized intensity distributions of target, numerical and experimental holographic images of sample 1 (“ZJU”) and 2 (“eagle”); scale bar, 50 μm.

For the experimental demonstration, we fabricate two samples, which differ in holographic image (“ZJU” for sample 1 and “eagle” for sample 2) and share the same far-field radiation distribution. More details about the fabrication process are provided in “Experimental Section”). Figure 3(a) and (b), respectively, show the optical micrograph and scanning electron microscopy (SEM) images of sample 1. Figure 3(c) schematically depicts the optical setup for capturing the holographic images. First, the linearly polarized 1,064 nm collimated laser beam passes through a neutral density filter and is cropped by a pinhole aperture (500 μm). A linear polarizer and a half-wave plate are used to produce the *x*-polarized light (red arrow), which is subsequently directed through a 2:1 beam reduction system to illuminate the metasurface. Finally, the holographic images are captured by a high-performance short-wave infrared CCD via an objective lens (40×, *NA* = 0.65) and a tube lens (*f* = 180 mm). Figure 3(d) shows the intensity distributions of the target, numerical and experimental holographic images of the two samples, which are all normalized to their maximum intensities, respectively. The captured images exhibit clarity and high contrast, despite minor disturbances in intensity uniformity caused by speckle noise in optical systems. To evaluate image quality, we compare the correlation coefficients of the numerical and experimental results with the target image, yielding values of 0.89 and 0.90 for sample 1 (the first row), and 0.86 and 0.84 for sample 2 (the second row), determining good performance of the designed holograms. Additionally, we further analyze the relationship between the experimental image quality and polarization angles by rotating the polarization of incident light, as shown in Figure S4 (Supplementary Material).

**Figure 4:**
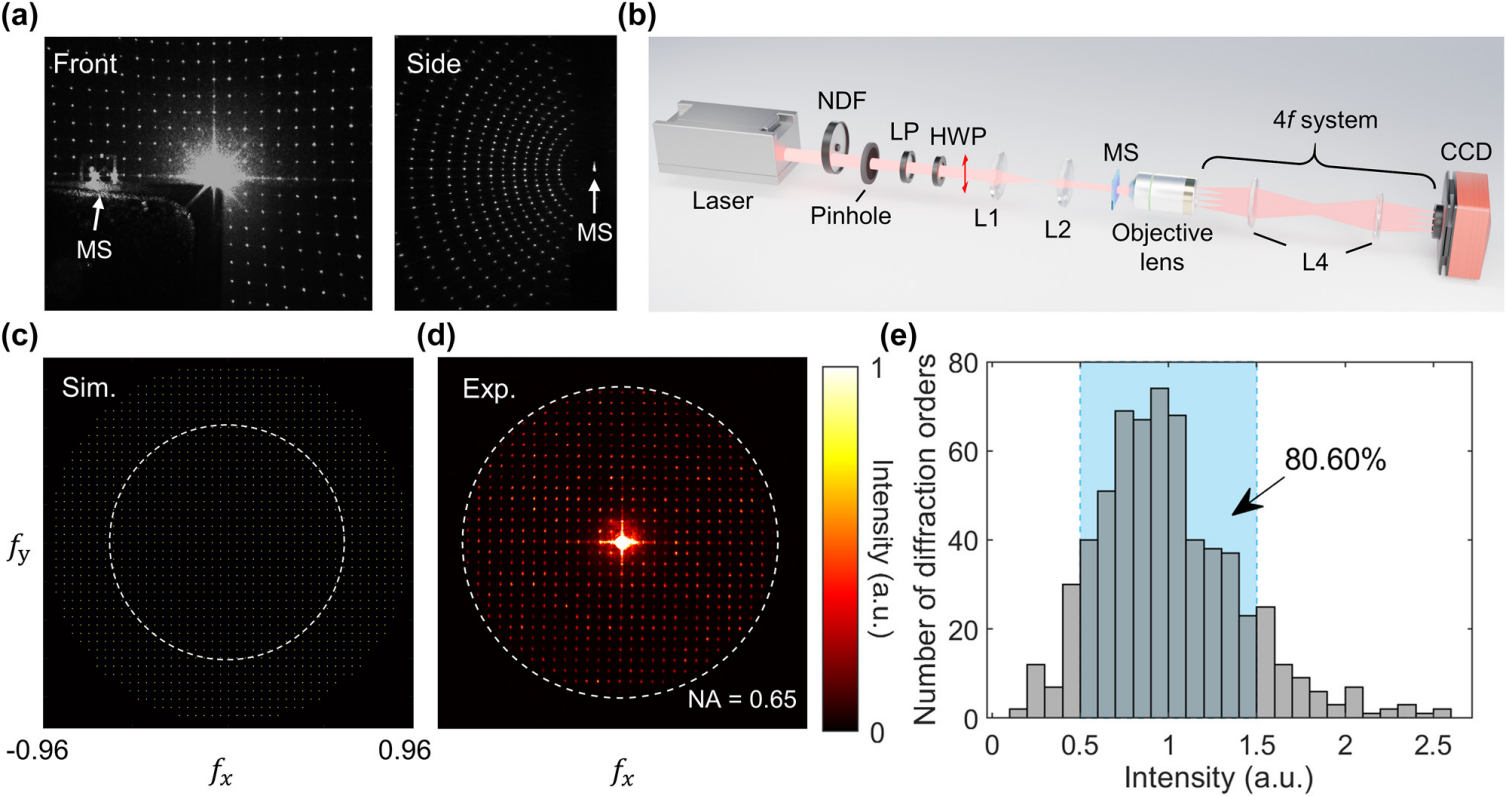
Experimental and numerical results of the projected spots. (a) Front and side view of the nearly half-space projection. MS, metasurface. (b) The optical setup for Fourier imaging of diffraction orders. The HWP is rotated to produce a *y*-polarized light (red arrow). L4, lens (*f* = 100 mm), a 4*f* lens system for *k*-space imaging. (c) Simulation and (d) experimental results of the diffraction orders. The dashed white circle represents the upper limit of captured diffraction orders induced by the NA of objective lens. (e) Counts of the diffraction orders against the intensity levels. The intensity is normalized to its average (target intensity). The bluish region covers the normalized intensity levels of 0.5∼1.5, where ∼80 % spots are concentrated here.

Next, we focus on the experimental performance of the far-field projection. Overall 1,513 spots are projected into the far field, with a maximum diffraction order of ±21 along the *x*- and *y*-axes, nearly covering the entire 2π transmissive space. Figure 4(a) shows the captured image of spots projected on the front and side observation planes of metasurface, demonstrating a ∼180° FOV of the spot cloud. The DE is calculated to be ∼59 %, measured by the intensity of all transmitted light normalized to that of the incident light traveling before the sample using a highly sensitive optical power meter. The zeroth order efficiency (ZOE), defined as the intensity of the zeroth order light normalized to that of the incident light, is measured to be 19 %, which is higher than the numerical value of 0.069 % (approximately equals to the target normalized intensity of 0.066 %). Methods for suppressing the ZOE are further presented in the Discussion Section.

Figure 4(b) depicts the Fourier space imaging setup for capturing the far-field diffraction orders. The half-wave plate is rotated to generate a *y*-polarized incident light (red arrow), and a 4*f* system is set up between the objective lens (40×, *NA* = 0.65) and the CCD to capture the *k*-space image. Figure 4(c) and (d) show the normalized intensity distributions of the simulation and experimental results, respectively. Since the observed FOV is limited by the NA of the objective lens, we can only capture diffraction orders under the condition of $\left(f_{x}^{2}+f_{y}^{2}\right)\leq {\left(\frac{NA}{\lambda }\right)}^{2}$ , as depicted in the white dashed lines in both figures. As a result, a total number of 629 diffraction orders are captured in Figure 4(d), with a calculated RMSE of 40.3 %, comparable to that of the previous study while we further broaden the FOV to ∼180° and raise the number of target spots by ∼22 times [38]. Section S5 (Supplementary Material) provides more details about the identification and intensity calculation of diffraction orders. Figure 4(e) shows the relative intensity distribution (normalized to the average intensity) against the number of diffraction orders. ∼80 % of the spot relative intensities are concentrated in the range of 0.5–1.5, showing a fairly uniform distribution. It should be noted here that the characteristics of the projected spots are not as ideal as the numerical calculations, specifically reflected in the relatively lower DE, stronger ZOE, and higher RMSE, which is mainly ascribed to the fabrication defects and phase deviations in numerical designs due to the ignorance of mutual coupling effects.

### 3.2 Experimental demonstration of depth perception driven by spot cloud projection

**Figure 5:**
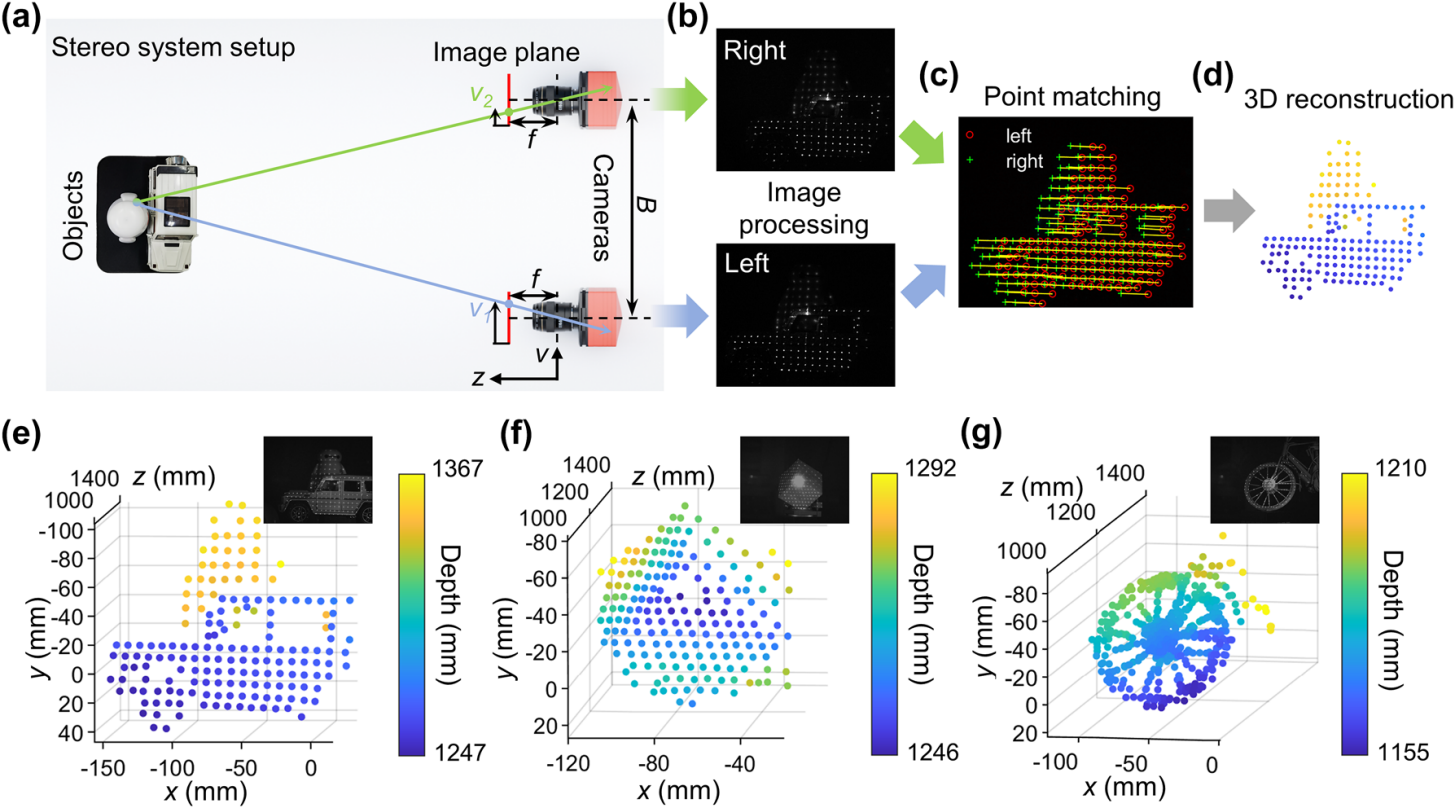
Experimental demonstration of the 3D reconstruction based on a stereo system. (a–d) Schematic of the flowchart of the 3D reconstruction scheme using a stereo system. (a) Top view of the stereo system setup. *B*, baseline; *f*, focal length; *v*, pixel ordinate; *z*, depth. (b) Example of an image pair captured by the stereo cameras and rectified using the calibrated parameters. (c) Schematic of the point matching algorithm. The red and green marks represent the feature points of the left and right image, and the yellow lines shows their one-to–one correspondence. (d) Schematic of the 3D reconstructed point cloud of object 1. (e–g) 3D reconstruction results of object 1 to 3; insets, reference pictures of the objects captured under the illumination of both the projected spots and another near-infrared flood lighting for clarity. (e) 3D reconstruction result of object 1 (models of a car and a pedestrian). (f) 3D reconstruction result of object 2 (inclined Rubik’s cube). (g) 3D reconstruction result of object 3 (wheel of a bicycle model). (e) And (f) are calculated when considering only the feature points extracted from the projected spots, while in (g) the whole images are used for feature points detection and matching to provide more points and details. Section S7 (Supplementary Material) presents the complete 3D reconstruction results.

To further exhibit its potential in depth perception, we perform an experimental demonstration of depth measurement based on the projected spot cloud. The experimental setup consists of three parts: the illumination from the metasurface, objects to be measured, and a stereo system. For illumination, the spot cloud generated by the proposed metasurface nearly covers the wholetransmissive space, while the divergence angle of each spot is suppressed by the interference of periodic arrangement (minimum divergence angle of 0.26°), thereby producing an illumination of relatively higher intensity and larger FOV compared to flood lighting. Meanwhile, the internal intensity distribution of each spot can provide extra feature information that helps to match the points captured in stereo cameras. A plano-convex lens (*f* = 2.5 mm) is added in front of metasurface at a certain distance to further adjust the spot density. For the objects, we perform the measurement of three different objects: object 1 is the models of a car and a pedestrian, object 2 is an inclined Rubik’s cube, and object 3 is the wheel of a bicycle model, which are placed at 45−65 cm away from the metasurface. Regarding the stereo system, it comprises two cameras separated by ∼50 mm, and is placed at ∼1.2 m away from the objects to ensure that the measured objects can be captured within the FOV of both cameras. Figure 5(a–d) schematically showcase the measurement flowchart from the stereo system setup to the final 3D reconstruction of object 1.

Before performing the depth measurements, stereo camera calibration is required to obtain precise parameters including the intrinsic and extrinsic parameters of both cameras using a standard checkerboard pattern. This establishes the mapping relationship from global coordinates to pixel coordinates, as well as the relative location of the two cameras (more details are provided in “Experimental Section”). After that, the images are captured by both cameras and rectified to avoid distortion and to align the pixel ordinates using the calibrated parameters, as Figure 5(b) exhibits. Feature points are then extracted and matched using the standard Kanade–Lucas–Tomasi (KLT) algorithm [46] (more details are provided in Section S6 in Supplementary Material), as shown in Figure 5(c). Finally, the depth of the matched feature point pair can be calculated using $Z=\frac{B\cdot f}{v_{1}-v_{2}}$ , where *B* is the distance between the cameras in the baseline, *f* is the focal length, and $\left(v_{1}-v_{2}\right)$ refers to the corresponding disparity. Figure 5(d) shows the schematic of the reconstructed object 1. For each projected spot, it can be characterized as a single point, thus giving us a point cloud of the target object.

Figure 5(e–g) successively exhibit the 3D reconstruction results of the three objects, where the insets show the pictures of the measured objects for reference. In Figure 5(e), the reconstruction of object 1 clearly distinguishes the car and pedestrian models; thus, capable for reconstructing separated object planes. The average lateral spatial resolution is 8.5 mm, and the maximum depth difference is 11.9 cm, as calculated by the difference between the maximum and minimum depths of points, which agrees well with the true depth of 12 cm. A denser spot cloud is projected on object 2 to show good reconstruction of continuously changing surface, as shown in Figure 5(f), where the average lateral spatial resolution of points on the front and left faces are 6.1 mm and 3.2 mm, respectively. It is noted that the relatively strong zeroth order light makes the region near it overexposed in the captured image, which leads to the absence of feature corners inside this region and forms a corresponding blind area in depth reconstruction consequently. By adjusting the lens to provide a denser spot cloud concentrated on object 3, thin structures in the wheel, including the rim of 8 mm and spokes as thin as 1.5 mm, can get a fairly good reconstruction attributed to the small divergence and high density of the projected spots. A total of 190 points are extracted from the 190 projected spots for calculation. The nonuniformity in intensity distributions inside and outside the spots, ascribed to the divergence of spots, speckles of scattered light in the optical system, and the illumination near the relatively strong zeroth order, can also provide us with extra information. Here, instead of extracting feature points only from the projected spots one-by-one, we input the whole image for corner detection and matching. As shown in Figure 5(g), overall 449 feature points are generated, showing fairly good morphology of thin structures reconstructed by the single-shot stereo system. The total time taken from reading the original image pair to generating the 3D coordinates of the point cloud is 0.058 s, corresponding to ∼17 fps real-time processing speed. All the original captured image pairs and 3D reconstruction results using only spot matching and whole image matching of all three objects are presented in Figure S7 (Supplementary Material).

## 4 Discussion

In the above, we propose a polarization-multiplexed metasurface that realizes high-quality holographic images and a uniform spot cloud with ∼180° FOV in two orthogonal linear polarization states. For holographic imaging, despite that the results of both the simulation and experiment have shown a strong correlation with the target image, the speckle introduced in the numerical calculations decides the upper limit of uniformity. As shown in Figure 3(d), compared to the target images, the numerically calculated images appear to be relatively dark after being normalized to their maximum intensity, thereby indicating intensity fluctuations brought by speckles of rather high intensity. To solve this problem, a new speckle-free optimizing method has been proposed recently to reduce the effect of speckles [47]. By narrowing the probability density distribution of the encoded phase to homogenize the optical superposition, holographic speckles can be largely removed, thus being capable of generating high-homogeneity, edge-sharp, and shape-unlimited holographic images.

For the spot cloud, the main problem here is the relatively higher FoM in experiment compared to that of simulations. Fabrication defects and ignorance of mutual coupling effects of adjacent meta-atoms are the two main reasons. The experimental performance can be improved with the development of fabrication technology and by adding the mutual coupling effects to numerical calculations. By designing the supercell with 4-fold rotational symmetry and applying vectorial electromagnetic simulation combined with interior-point method for optimization, more precise numerical results have been obtained for supercells containing a small number of unit-cells in previous studies [37], [38]. For a greater number of pixels, however, full-wave simulation of the whole supercell is infeasible. Instead, we can still use the scalar diffraction theory for optimization, while the phase of each meta-atom is revised by performing simulation containing the designed meta-atom itself and its adjacent meta-atoms [48]. Another challenge is the suppression of the relatively strong zeroth order signal, ascribed to the non-modulated zeroth order light and phase deviations caused by fabrication errors and ignorance of mutual coupling effects in numerical calculation. In addition to improving fabrication accuracy and using full-wave simulations or the improved scalar diffraction algorithm to take into account mutual coupling effects, as mentioned above, we can also adopt the Even-numbered Dammann gratings design [49] to eliminate the zeroth order light. Even-numbered Dammann gratings, which include a translational symmetry about the period midpoint with a corresponding phase offset of π, can largely suppress the zeroth order light sensitivity to the fabrication defects, and have been proved to perform higher DE and lower ZOE in experiments and real applications. In Section S8 (Supplementary Material), we design an even-numbered Dammann grating to suppress the zeroth order light while maintaining the FOV of ∼180°. The uniformity and DE are comparable to the odd-numbered type proposed in the main text, while the zeroth order light is largely suppressed, with a near-to-zero ZOE, and its robustness to phase deviations is largely improved. Although this even-numbered design contains less spots and lower angular resolution, ascribed to the elimination of even-numbered diffraction orders, these characteristics can be improved simply by increasing the period of supercells.

In this work, we perform a stereo depth measurement to exhibit the potential of our proposed metasurface in 3D spatial sensing. Attributed to its large FOV and number of projected spots, it is promising to produce a miniaturized and compact solid-state system with a single shot. To further shrink the size of the projection and stereo vision system, a metalens-assisted driving vision scheme has been proposed recently [50]. Other coded spot cloud can be applied to further enhance the features of images and manage to obtain 3D reconstruction by matching the points with reference images captured by a single camera [51], [52]. Besides, along with a pulsed light source for illumination and single photon avalanche diode (SPAD) array for depth detection, it can be integrated into a time-of-flight (ToF) 3D sensing device. Compared with bulky scanning devices, the proposed solid-state device has much more compact size, longer lifetime, and higher frame rate [5], [53]. Its lacuna in long-range detection is ascribed to the relatively low power of spot introduced by light splitting, which can be counteracted by using high-power laser or laser array (such as vertical-cavity surface-emitting laser array) and by increasing the number of supercells to further narrow down the divergence and concentrate the power of the projected spots. Regarding the characterization of the multifunctionality, a comprehensive comparison with multifunctional metasurfaces proposed in recent representative papers is presented in Table S2 (Supplementary Material). To further enhance the multiplexing potential of miniaturized multifunctional metasurfaces, integration of more functions is expected by extending the channels of polarization states, for example, orbital angular momentum can provide orthogonal multi-channels [54]. In addition, encoding more multiplexing dimensions, such as wavelength [55], twisted angle [56], surrounding medium [57], etc., can greatly increase the multiplexing capacity, while combining methods like engineered noises [28], phase compensation mechanism [58], [59], machine learning assisted schemes [55], etc., can help to suppress the crosstalk between channels and decrease each efficiency loss.

## 5 Conclusions

In conclusion, we propose a function-switchable metasurface that generates holographic images in the Fresnel region for display, and a uniform spot cloud projected into the far field for 3D spatial sensing via polarization multiplexing. The experimentally captured holographic image shows high image quality and sharpness, with a correlation coefficient up to ∼0.9. Meanwhile, the spot cloud contains over 1.5 K spots and FOV of ∼180°, which is capable of illumination and depth measurement for objects located in the entire transmissive space. A 3D reconstruction experiment has been carried out based on a stereo system, in which all the separated objects, continuously varying surfaces and objects containing thin structures are well reconstructed, showing good performance in 3D spatial sensing. Toward promising to achieve highly integrated and compact system, the proposed metasurface design paves a way for miniaturized solid-state optical devices for display and 3D spatial sensing, especially prospective in cutting-edge technologies such as AR/MR technologies, intelligent driving, and robot vision.

## 6 Experimental section

### 6.1 Fabrication process

First, we used the electron beam evaporation tool to deposit 600 nm thick amorphous silicon on a 500 μm thick fused silica substrate. Next, a 270 nm thick positive tone photoresist of polymethyl meth-acrylate (PMMA, AR-P 679.04) was spin-coated on the sample at 4,000 r.p.m. for 60 s and baked at 150 °C for 10 min. A conductive polymer (AR-PC 5092.02) was spin-coated at 4,000 r.p.m. for 30 s and baked at 120 °C for 2 min to prevent charging effects. Then the designed patterns were written on the PMMA using a standard electron beam lithography (EBL). After exposure, the conductive layer was removed in deionized water and the PMMA was developed in MIBK/IPA 1:3 solution for 35 s and rinsed by IPA for 35 s. Next, a 20 nm thick chromium (Cr) was deposited on the resist by sputtering and patterned as an etching mask after lift-off in acetone. Using inductively coupled plasma (ICP) etching technology, the patterns were transferred to the silicon layer. The remaining Cr mask was removed by Cr etchant.

### 6.2 Depth reconstruction

First, we used MATLAB Camera Calibrator to calibrate the intrinsic parameters (the focal length, optical center, skew parameter) of each camera. A standard checkboard pattern was placed at different angles and locations to cover the entire camera field for better calibration. Then the calculated intrinsic parameters of the two cameras were directly input to the MATLAB Stereo Camera Calibrator, where the extrinsic parameters (the relative rotation and translation) could be retrieved after rectifying 20−30 pairs of images captured by the stereo cameras. This helped to improve the accuracy of parameter optimization and to reduce the deviations of the intrinsic parameters caused by incomplete field coverage when directly using the latter calibrator. Incomplete or high-error images were excluded to improve the optimization. After calibration, image pairs of spots projected on the objects were captured by the stereo cameras and matched using the KLT algorithm in MATLAB. Finally, the point cloud coordinates were calculated using $Z=\frac{B\cdot f}{v_{1}-v_{2}}$ .

## Supporting information

Supporting information is available in Supplementary Material including: FoM for both functions. Simulated phase and amplitude of different unit-cells and the final matched phase and amplitude deviations in both polarization states. Comparison of numerical key parameters using different encoding methods. Relation between the experimental image quality and polarization angles. Calculation of the intensity of diffraction orders. Expression of KLT algorithm. Details of 3D reconstruction of the three objects. Design of an even-numbered Dammann grating. Comparison of the multifunctionality among different metasurfaces.

## Supplementary Material

Supplementary Material Details
