# Supplementary material for: Polarization-controlled metasurface for simultaneous holographic display and three-dimensional depth perception
Source: Nanophotonics. 2025 Jan 17;14(2):197–207. doi: 10.1515/nanoph-2024-0509 (PMC11806502; doi:10.1515/nanoph-2024-0509)
Supplement: Supplementary file 1 — Supplementary Material Details [file j_nanoph-2024-0509_suppl_001.docx]

Supporting Information

Polarization-controlled metasurface for simultaneous holographic display and three-dimensional depth perception

Shuhan Guo^1^, Yifan Shao^1^, Junjie Zhan^1^, Jiaqi Yu^1^, Yubo Wang^1^, Pankaj K. Choudhury^1^, Hugo E. Hernandez-Figueroa^2^ and Yungui Ma^1,^*

^1^State Key Lab of Modern Optical Instrumentation, Centre for Optical and Electromagnetic Research, College of Optical Science and Engineering; International Research Center for Advanced Photonics (Haining Campus), Zhejiang University, Hangzhou 310058, China

^2^ Department of Communications, School of Electrical and Computer Engineering (FEEC), University of Campinas (UNICAMP), Cam´pinas 13083-852, São Paulo, Brazil

[*yungui@zju.edu.cn](mailto:*yungui@zju.edu.cn)

Number of pages: 11

Number of figures: 8

Number of tables: 2

Section S1. Figure-of-merit.

Both functions are optimized separately using the GS algorithm. The FoM of holography is set as the Pearson correlation coefficient, while the FoM of Dammann grating is set as $\text{FoM}\text{ }\text{=}\text{ }\text{(1}\text{ }\text{-}\text{ }\text{DE)}\text{ }\text{+}\text{ }\text{RMSE}$.


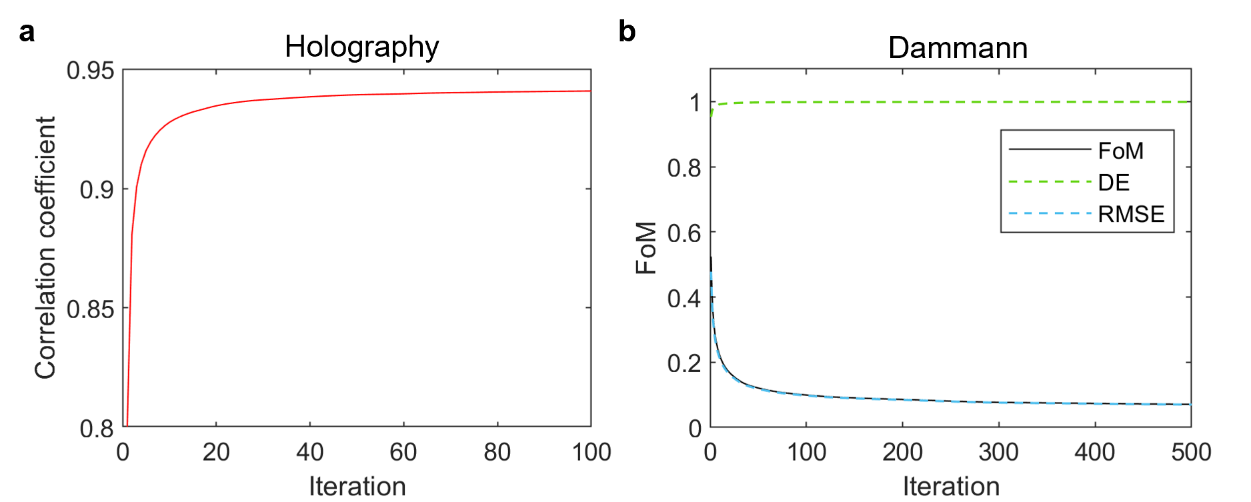


**Figure S1.** Plots of FoM as a function of iteration. (a) FoM of holography. (b) FoM of Dammann grating.

Section S2. Design of the unit-cell structure.

**
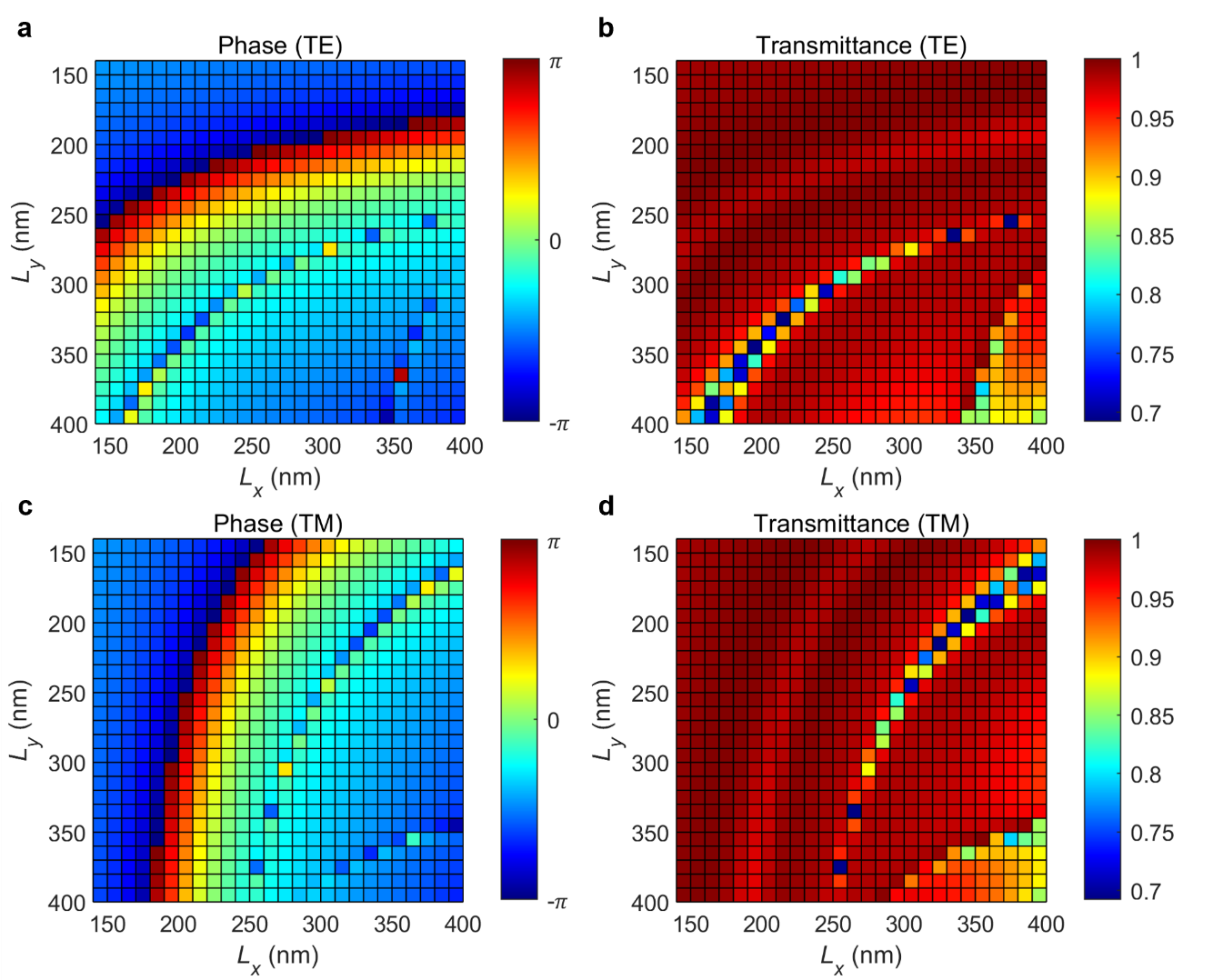
**

**Figure S2.** Simulated phase and transmittance of a plane wave passing through the 2D unit-cell with respect to *L_x_* and *L_y_*. (a,b) Phase and transmittance induced by the incident light of TE mode (*y*-polarized). (c,d) Phase and transmittance induced by the incident light of TM mode (*x*-polarized). The height and period of the Si nanorod is fixed to 600 nm and 520 nm, respectively. The step size of scanning *L_x_* and *L_y_* is 10 nm.

Section S3. Matched phase deviations.

The nanostructure of each pixel is retrieved by satisfying the minimum difference of complex amplitude between the optimized phase profile of both polarization states and the simulated results in structure library. As a result, the absolute average phase and amplitude errors are, respectively, ~0.094 rad and ~0.015 per pixel for both polarization states, showing a rather good matching and function balance.


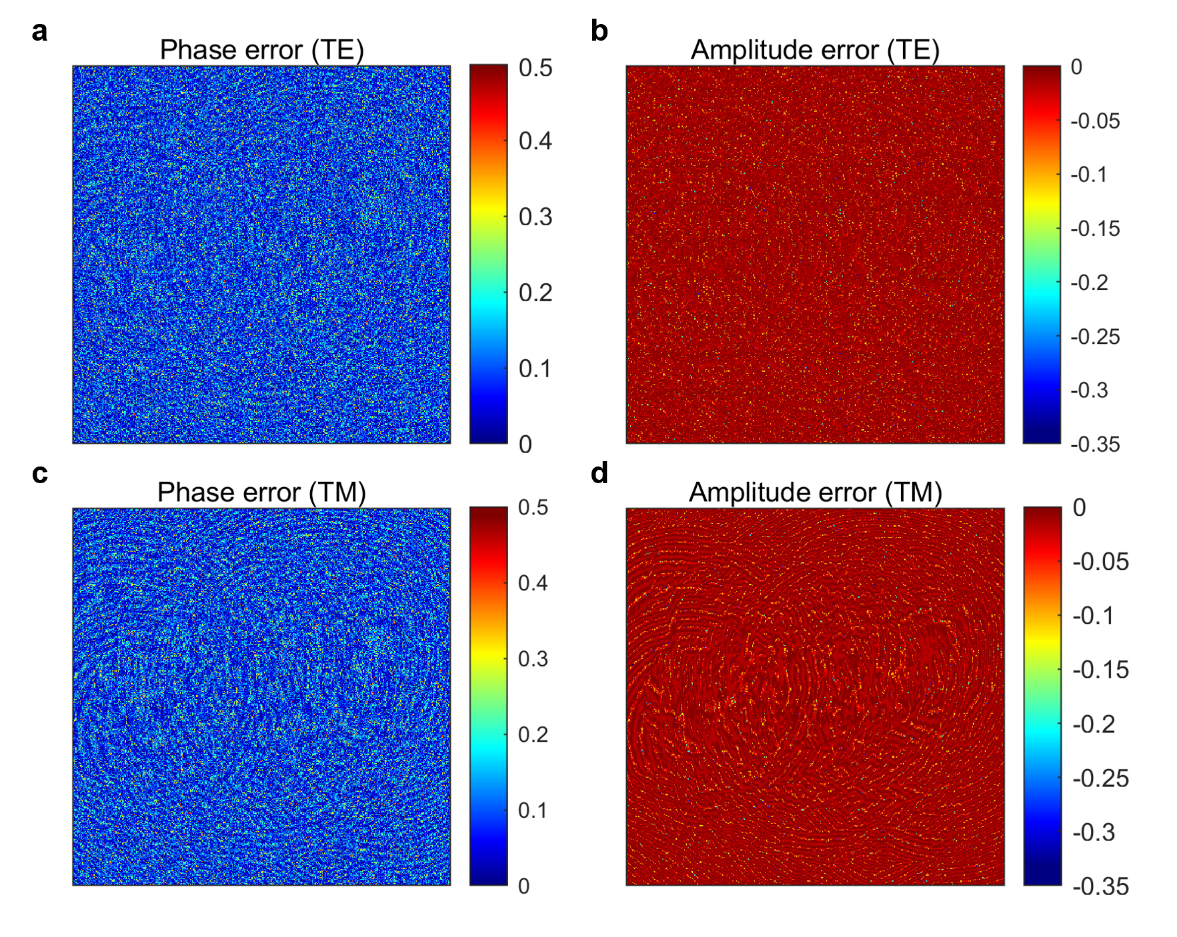


**Figure S3.** Matched complex amplitude deviations of the metasurface. (a,b) Phase and amplitude profile deviations in the TE mode. (c,d) Phase and amplitude profile deviations in the TM mode.

Section S4. Comparison of numerical key parameters using different encoding methods.

By maintaining the same design parameters (wavelength, period, pixel numbers, NA constraints, iteration times, etc.) and regarding the same design aims, we compared the differences in results using different encoding methods. Table S1 shows the performance of numerical key parameters when encoding only a single function, encoding both functions using polarization-independent dual-loop GS algorithm [1], and encoding both functions using polarization multiplexing (this work), respectively. The specific parameters comprise the Pearson correlation coefficient *ρ* for holography, the diffraction efficiency (DE) and the root-mean-square error (RMSE) for Dammann gratings, and the total FoM loss calculated by summing the deviations between each of the three parameters and the corresponding parameters when operating in a single-function encoding mode. The polarization multiplexing method introduces a negligible efficiency loss compared to those encoding only a single function, due to phase matching errors brought by meta-atoms (small average phase error of ~0.094 rad and amplitude error of ~0.015 per pixel for both polarization states). It is much lower than that using the polarization-independent multiplexing method proposed in Ref. [1], showing a better working performance and greater potential for multi-channel multiplexing.

| *Numerical Key Parameters* | *Only the Holographic Image* | *Only the Dammann Gratings* | *Both Functions Using Polarization-Independent Position Multiplexing ([1])* | ***Both Functions Using Polarization and Position Multiplexing (this work)*** |
| --- | --- | --- | --- | --- |
| *ρ* | 0.937 | / | 0.835 | **0.937** |
| DE | / | 99.9% | 99.7% | **98.3%** |
| RMSE | / | 5.9% | 23.1% | **7.1%** |
| FoM Loss | / | / | 0.276 | **0.028** |

Tab. S1: Comparison of numerical key parameters using different encoding methods

Section S5. Relation between the experimental image quality and polarization angles.

By rotating the half-wave plate, we manage to get series of linearly polarized light with different polarization angles (step size: 10°). Figure S4 presents the corresponding captured images of “eagle” and “ZJU” within the blue and red borders, respectively. Figure S4b illustrates the relation between the correlation coefficient and rotated polarization angles with respect to the *x*-polarization. For rotated polarization angle below 60°, the reconstructed images appear to be strongly correlated with the target one ($\text{ρ}\text{ }\text{>}\text{ }\text{0.8}$). As this angle increases, the correlation coefficient drops dramatically, and appears to be extremely weakly correlated ($\text{ρ}\text{ }\text{<}\text{ }\text{0.2}$), when it comes to its orthogonal polarization state. This proves weak crosstalk between the two orthogonal channels.


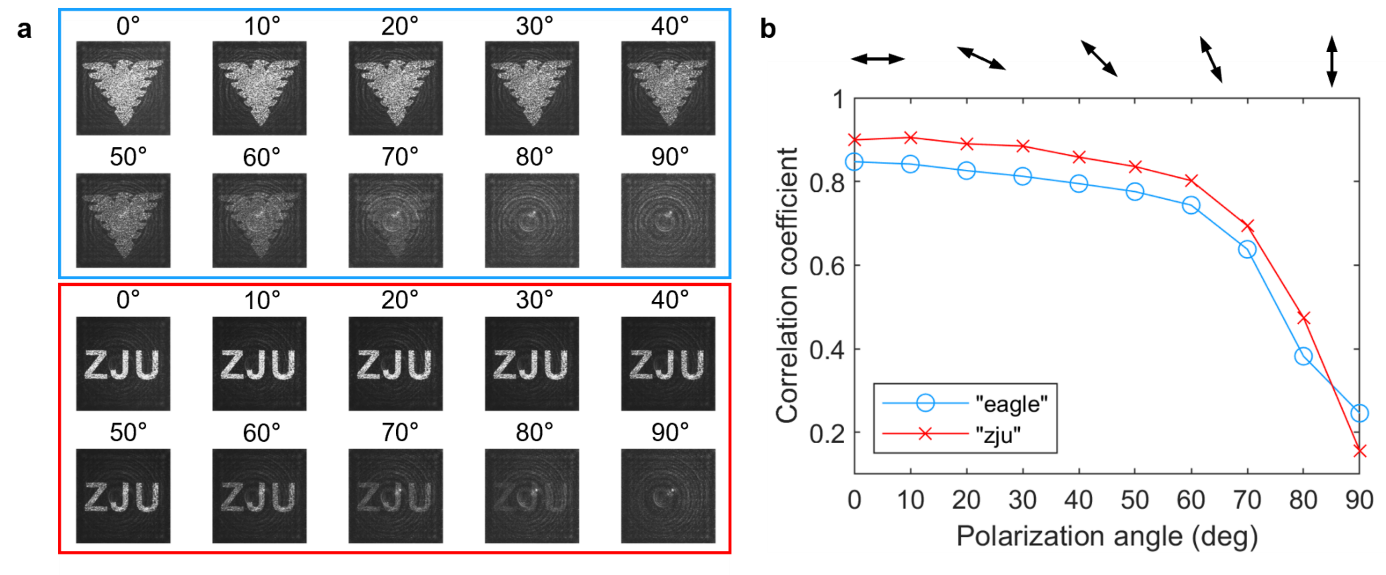


**Figure S4.** Relation between the experimental image quality and polarization angles. (a) Captured images illuminated by the lights of different polarization angles (0~90° with respect to the *x*-direction). Inside the blue and red borders are the images of “eagle” and “ZJU”, respectively. (b) The plot of correlation coefficient as a function of the rotated polarization angles.

Section S6. Calculation of the intensity of diffraction orders.

Figure S5 shows the numerical and experimental results of diffraction orders, which are recognized using the 2D difference method and marked within the area outlined in white. A 40× objective lens with an NA of 0.65 is used in experiment to capture diffraction orders satisfying $\text{(}{\text{f}_{\text{x}}}^{\text{2}}\text{ }\text{+}\text{ }{\text{f}_{\text{y}}}^{\text{2}}\text{)}\text{ }\text{≤}\text{ }{\text{(}\frac{\text{NA}}{\text{λ}}\text{)}}^{\text{2}}$. The intensity of each order is calculated by integrating the gray value over the spot region, and then normalized to the total intensity of all orders calculated. To calculate the corresponding RMSE, the overexposed low diffraction orders are not considered here due to the unreliable data owing to the relatively strong zeroth-order light. Consequently, a total number of 629 diffraction orders are calculated, with an RMSE of 40.3%. The experimental RMSE is higher than the simulation result of 7.1%, which is mainly ascribed to phase deviations caused by fabrication defects and ignorance of coupling effects in numerical calculations.


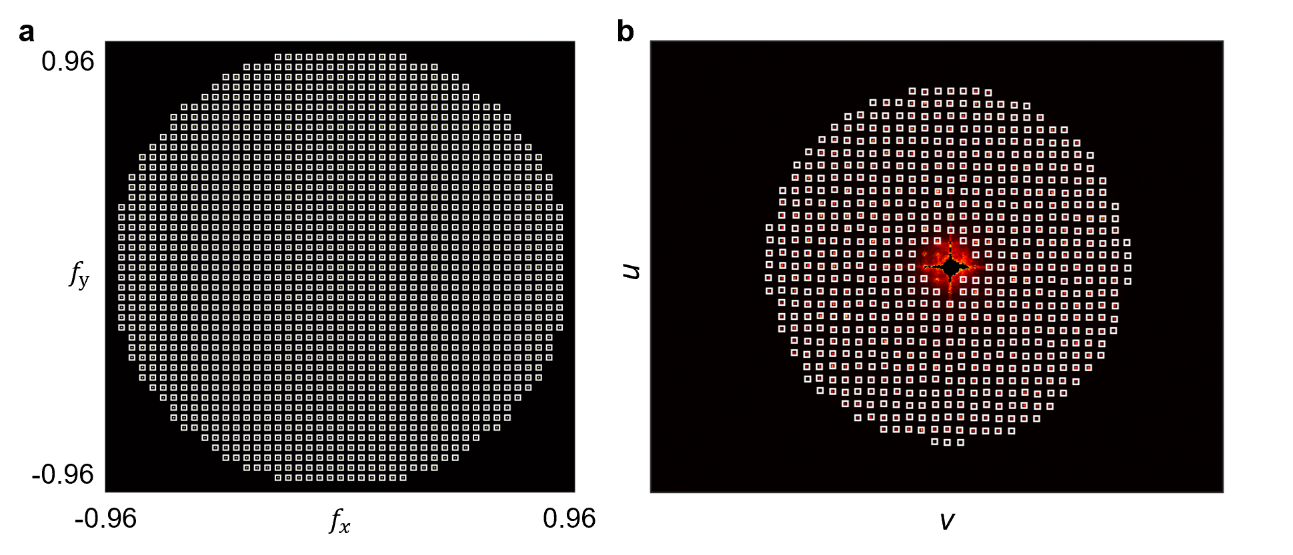


**Figure S5.** Calculation of the intensity of diffraction orders. (a) Numerical result of all diffraction orders. (b) Experimentally captured diffraction orders using an objective lens (NA = 0.65). The positions of diffraction orders are marked with white borders.

Section S7. KLT algorithm.

KLT algorithm is an optical flow method in target tracking [2]. It is applied under three conditions – (i) constant brightness, (ii) continuous frames or small spatial displacement, and (iii) same displacement of points inside the calculated sub-window. The feature points (corners) are first recognized in the first image (or frame, for video); then each one is estimated by translation or affine to find the corresponding vector of each feature point between images to realize tracking in following images. Specifically, in every calculated sub-window *W* of two images, the points share the same displacement (*d_x_*, *d_y_*). Thus, tracking the sub-window requires minimizing the difference of it between the two images, which is expressed as

$$\begin{aligned} \text{ϵ}\text{ =}\iint_{\text{W}} \left[ \text{J}\left( \text{x }\text{+ }\frac{\text{d}}{\text{2}} \right) \text{- }\text{I}\left( \text{x }\text{- }\frac{\text{d}}{\text{2}} \right) \right]^{\text{2}}\text{w}\left( \text{x} \right)\text{d}\text{x}\#\left( S1 \right) \end{aligned}$$

where *J* and *I* represent the gray value of two images, and $w(x)$ is the weight function. To minimize Equation S3, its derivative should be zero, which is

$$\begin{aligned} \frac{\text{∂}\text{ϵ}}{\text{∂}\text{d}}\text{ }\text{=}\text{ }\text{2}\iint\left[ \text{J}\left( \text{x}\text{ }\text{+}\text{ }\frac{\text{d}}{\text{2}} \right) \text{-}\text{ }\text{I}\left( \text{x}\text{ }\text{-}\text{ }\frac{\text{d}}{\text{2}} \right) \right]\left[ \frac{\text{∂}\text{J}\left( \text{x}\text{ }\text{+}\text{ }\frac{\text{d}}{\text{2}} \right)}{\text{∂}\text{d}} \text{-}\text{ }\frac{\text{∂}\text{I}\left( \text{x}\text{ }\text{-}\text{ }\frac{\text{d}}{\text{2}} \right)}{\text{∂}\text{d}} \right]\text{w}\left( \text{x} \right)\text{d}\text{x}\text{ }\text{=}\text{ }\text{0}\#\left( S2 \right) \end{aligned}$$

Due to the Taylor expansion,

$$\begin{aligned} \text{J}\left( \text{ε} \right)\text{ }\text{≈}\text{ }\text{J}\left( \text{α} \right)\text{ }\text{+}\text{ }\left( \text{ε}_{\text{x}\text{ }}\text{-}\text{ }\text{α}_{\text{x}} \right)\frac{\text{∂}\text{J}}{\text{∂}\text{x}}\left( \text{α} \right)\text{ }\text{+}\text{ }\left( \text{ε}_{\text{y}} \text{-}\text{ }\text{α}_{\text{y}} \right)\frac{\text{∂}\text{J}}{\text{∂}\text{y}}\left( \text{α} \right)\#\left( S3 \right) \end{aligned}$$

The derivative is rewritten as

$$\begin{aligned} \frac{\text{∂}\text{ϵ}}{\text{∂}\text{d}}\text{ }\text{=}\iint\left[ \text{J}\left( \text{x} \right) \text{-}\text{ }\text{I}\left( \text{x} \right)\text{ }\text{+}\text{ }\text{g}^{\text{T}}\text{d} \right]\text{g}\text{(}\text{x}\text{)}\text{w}\left( \text{x} \right)\text{d}\text{x}\text{ }\text{=}\text{ }\text{0}\#\left( S4 \right) \end{aligned}$$

where

$$\begin{aligned} \text{g}\text{ }\text{=}\text{ }\left[ \frac{\text{∂}}{\text{∂}\text{x}}\left( \frac{\text{I}\text{ }\text{+}\text{ }\text{J}}{\text{2}} \right)\text{ }\frac{\text{∂}}{\text{∂}\text{y}}\left( \frac{\text{I}\text{ }\text{+}\text{ }\text{J}}{\text{2}} \right) \right]^{\text{T}}\#\left( S5 \right) \end{aligned}$$

Equation S6 can be further written as

$$\begin{aligned} \text{Zd}\text{ }\text{=}\text{ }\text{e}\#\left( S6 \right) \end{aligned}$$

where

$$\begin{aligned} \text{Z}\text{ }\text{=}\iint\text{g}^{\text{T}}\left( \text{x} \right)\text{g}\left( \text{x} \right)\text{w}\left( \text{x} \right)\text{d}\text{x}\#(S7) \end{aligned}$$

$$\begin{aligned} \text{e}\text{ }\text{=}\iint\text{[}\text{I}\left( \text{x} \right) \text{-}\text{ }\text{J}\text{(}\text{x}\text{)]}\text{g}\left( \text{x} \right)\text{w}\left( \text{x} \right)\text{d}\text{x}\#(S8) \end{aligned}$$

To solve *d*, *Z* needs to satisfy the invertible matrix of $\text{Z}\text{ }\text{*}\text{ }\text{Z}^{\text{ }\text{T}}$. In general, the corner points have this characteristic, and can work as the feature points, which are then put in an iterative calculation of the incremental translation after alignment with the given target template. Figure S6 gives an example of feature point matching.


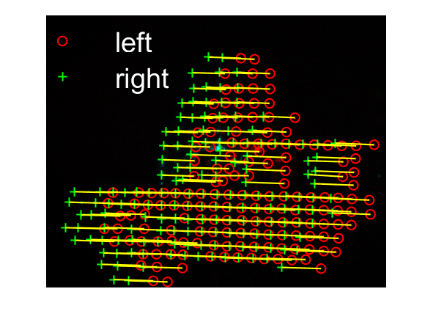


**Figure S6.** Feature point matching. The red and green marks are the matched points captured from the left and right cameras, respectively. The yellow line depicts the point-to-point matching.

Section S8. Original image pairs and the corresponding 3D reconstruction results.


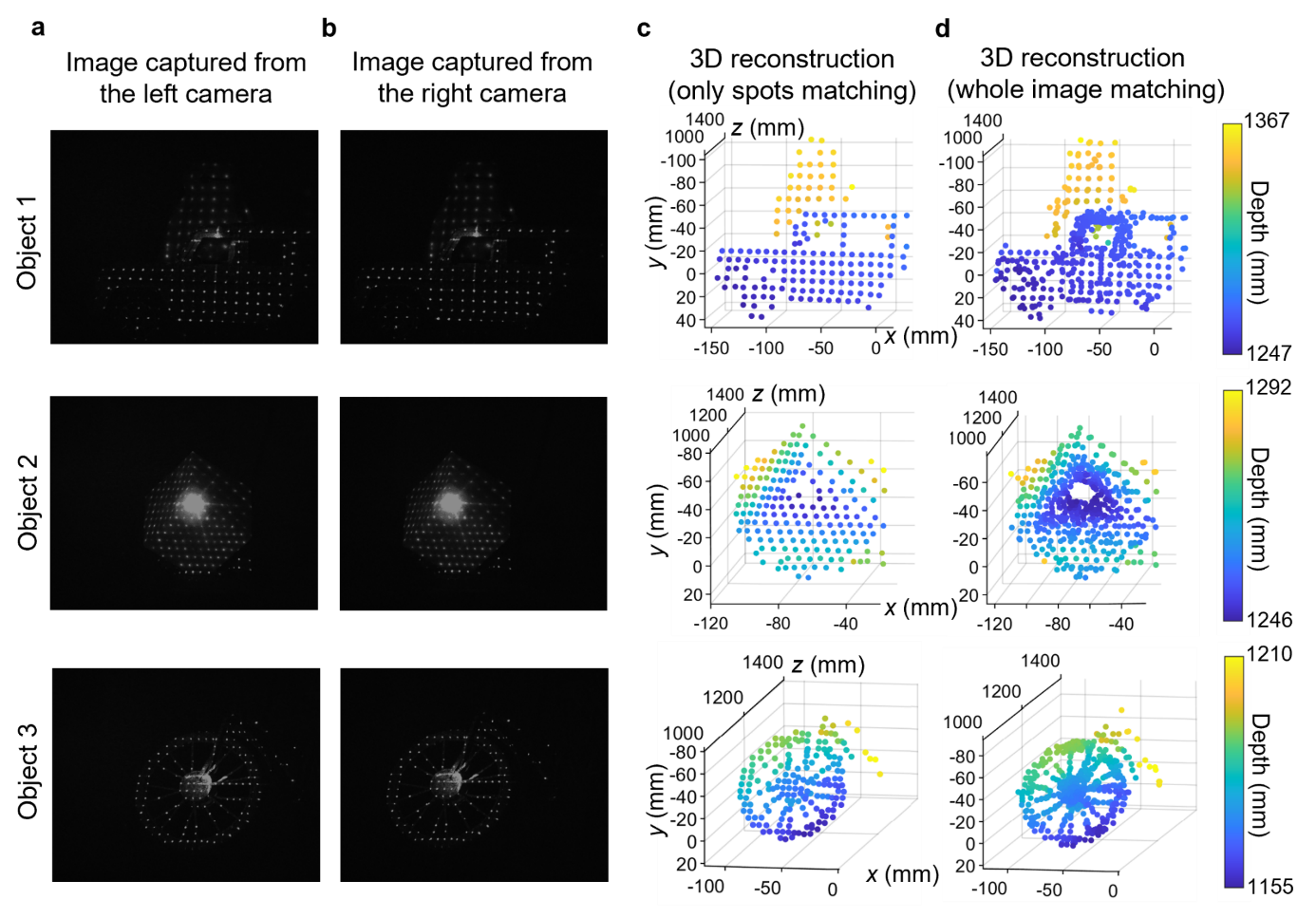


**Figure S7.** Original image pairs and the corresponding 3D reconstruction results. (a,b) The original image pairs of the three objects captured by stereo cameras. (c) 3D reconstruction results when only each projected spot is regarded as a feature point for reconstruction. This shows the arrangement of the projected spot array, and is consistent with the ToF scheme using SPAD array detector. (d) 3D reconstruction results when the feature points are detected and matched through whole image. The background light from environment, the relatively strong zeroth order light, the speckle of scattered light in the optical system, and the divergence of projected spots, can all induce intensity disturbance. Thus, besides the projected spots, feature points (corner points) can also be extracted from the region where the intensity distribution of the local light field is non-uniform.

Section S9. Even-numbered Dammann gratings.

Even-numbered Dammann gratings were first proposed by Rick L. Morrison in 1991 [3]. By designing the phase profile to be translational symmetric about the period midpoint with a corresponding phase offset of π, the even-numbered diffraction orders, including the zeroth order, can be deeply suppressed.

A similar design composed of 44 × 44 pixels per supercell is discussed in Figure S8. Figure S8a shows the retrieved translational symmetric phase profile. Figure S8b depicts the far-field intensity distribution, where a total number of 392 odd-numbered diffraction orders are optimized to be uniform, also nearly covering the entire transmissive space. Figure S8c illustrates its corresponding FoM as a function of iteration, with a DE of 99.9% and RMSE of 6.6%, comparable to the FoM of the odd-numbered design (proposed in the main text), whereas the ZOE is near-to-zero (ZOE = 1.5*10^−33^).

Next, we calculate their robustness to phase deviations brought by fabrication defects and numerical calculations. Phase deviation can be expressed as $\text{φ}_{\text{err}}\text{ }\text{=}\text{ }\text{w}_{\text{err}}\text{ }\text{*}\text{ }\text{rand}\left( \text{M}\text{,}\text{ }\text{N} \right)\text{ }\text{*}\text{ }\text{2π}$, where$\text{w}_{\text{err}}$is the weight of random error function, and $\text{rand}\text{ }\text{(}\text{M}\text{,}\text{ }\text{N}\text{)}$ is the array of random numbers evenly distributed between (0, 1), where *M* and *N* refer to the pixel numbers per supercell. We increase $\text{w}_{\text{err}}$from 0 to 1 with a step size of 0.1, while for each step, up to 2000 different random arrays are input as the random error and be added to the retrieved phase profile of the supercell to calculate both the RMSE and ZOE. The average results are output as the final results for each $\text{w}_{\text{err}}$. As shown in Figure S8d, both the RMSE and ZOE of the even-numbered design increase more slowly than those of the odd-numbered design, indicating better performance of the even-numbered Dammann gratings in experiments and applications in real scenarios.


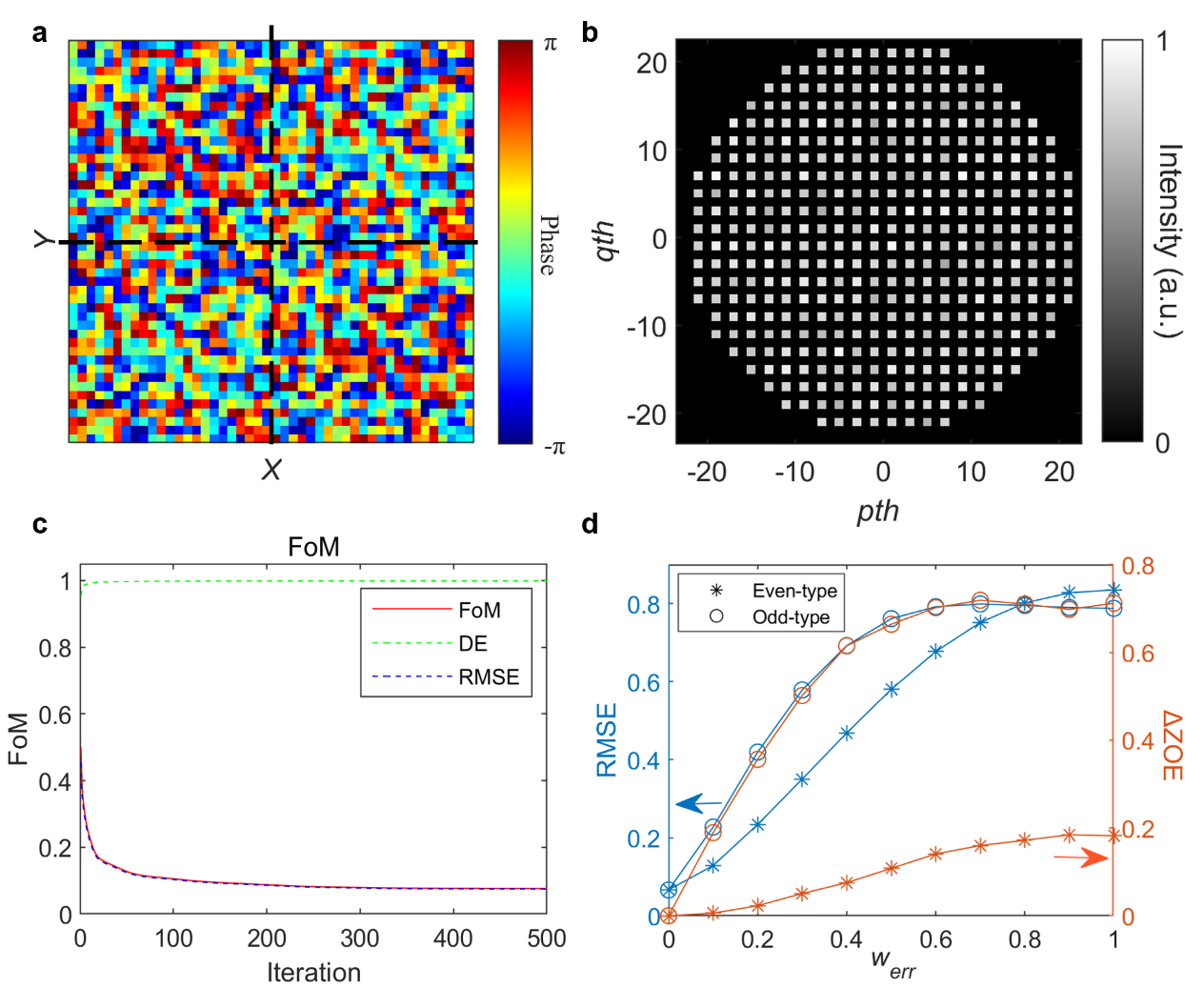


**Figure S8.** Even-numbered Dammann gratings. (a) Phase profile of the even-numbered design. The horizontal and vertical dashed black lines show the axis of symmetry. (b) Far-field intensity distribution against the diffraction order (*p*th, *q*th). The even-numbered diffraction orders are deeply suppressed. (c) FoM as the function of iteration. (d) Robustness to phase deviations. The blue lines refer to RMSE, whereas the orange lines refer to ΔZOE, defined as $\text{∆ZOE}\text{ }\text{=}\text{ }\text{(ZOE}\text{ }\text{-}\text{ }\text{ZOE}_{\text{0}}\text{)}\text{ }\text{/}\text{ }\text{I}_{\text{aim}}$, with ZOE_0_ being the original ZOE without adding any phase error, and *I_aim_* being the aimed intensity for each diffraction order. The even-numbered design shows a more soothing rise compared to the odd-numbered design, thereby indicating better robustness to the phase deviations.

Section S10. Comparison of the multifunctionality among different metasurfaces.

Tab. S2: Comparison of the multifunctionality among different metasurfaces

| **Multifunctional Metasurfaces** | **Specific Functionalities** | **Meta-Structures** | **Multiplexing Method** | **Experimental Efficiency** |
| --- | --- | --- | --- | --- |
| Ref. [4] | Multi-channel Fresnel holography | Composite MIM nanobricks | Polarization and position multiplexing | Maximum energy ditribution of 55% and maximum correlation of 0.8 for 5 channels |
| Ref. [5] | On-chip/free space focusing or holography | Composite dielectric nanobricks | On-chip/free space and polarization multiplexing | Focusing Efficiency of 0.5%/10.4%; Holographic efficiency of 3.5%/30% |
| Ref. [6] | Focusing/de-focusing/ twisted light wavefront | V-shaped metallic nanoantennas | Co- and cross- polarization multiplexing | 0.08% and 0.21% for the scattered *E_x_/E_y_* components |
| Ref. [7] | Focusing/holography | Composite MIM nanobricks | Frequency and polarization multiplexing | Fousing efficiency of 10.2%~59.2% for 4 frequencies and 2 polarization states |
| Ref. [8] | Multi-channel holography | Composite MIM nanobricks | Surrouding mediums and wavelength multiplexing | 7.61/ 15.46% in the air/oil; 11.47%~13.48% in four wavelengths |
| Ref. [9] | Multi-channel holography | Dielectric nanoposts | OAM multiplexing | 0.48~0.64 for 4 OAM channels |
| Ref. [10] | Multi-channel holography | Dielectric nanobricks | Twisted angle multiplexing | Numerical correlation around 0.8577 for 2-layer metasufaces |
| This work | Holography/depth perception | Elliptic dielectric nanobricks | Polarization and position multiplexing | Correlation of 0.9 for holography; Diffraction efficiency of 59% and RMSE of 40.3% for Dammann gratings |

References

1. S. Guo et al., "Multifunctional Metasurface: Holography and Spot Cloud Projection," *Adv. Opt. Mat.*, vol. 12, no. 28, p. 241235, 2024.
2. B. D. Lucas and T. Kanade, "An Iterative Image Registration Technique with an Application to Stereo Vision," 1981 Int. Joint Conf. Artif. Intell., vol. 2, 1981
3. R. L. Morrison, "Symmetries that simplify the design of spot array phase gratings," *J. Opt. Soc. Am. A-Opt. Image Sci. Vis.*, vol. 9, no. 3, pp. 464-471, 1992.
4. B. Xiong et al., "Breaking the limitation of polarization multiplexing in optical metasurfaces with engineered noise," *Science*, vol. 379, no. 6629, pp. 294-299, 2023.
5. W. Li et al., "Multifunctional Metasurface for Simultaneous Light Manipulation under Both Guided-Wave and Free-Space Incidence," *ACS Photonics*, vol. 11, no. 4, pp. 1724-1733, 2024.
6. L. Deng et al., "Functionality Expansion of Guided Mode Radiation via On-Chip Metasurfaces," *Nano Lett.*, vol. 24, no. 29, pp. 9042-9049, 2024.
7. W. Ma et al., "Pushing the limits of functionality‐multiplexing capability in metasurface design based on statistical machine learning," *Adv. Mater.*, vol. 34, no. 16, p. 2110022, 2022.
8. B. Xiong et al., "Realizing colorful holographic mimicry by metasurfaces," *Adv. Mater.*, vol. 33, no. 21, p. 2005864, 2021.
9. H. Ren et al., "Metasurface orbital angular momentum holography," *Nat. Commun.*, vol. 10, no.1, p. 2986, 2019.
10. Z. Fan et al., "Holographic multiplexing metasurface with twisted diffractive neural network," *Nat. Commun.*, vol. 15, no. 1, p. 9416, 2024.
